# Toxicity of Povidone-iodine to the ocular surface of rabbits

**DOI:** 10.1186/s12886-020-01615-6

**Published:** 2020-09-01

**Authors:** Sunyoung Kim, Yongsun Ahn, Yeojin Lee, Hyunseung Kim

**Affiliations:** 1 Healing Eye Center, Seoul, Korea; 2 College of Medicine, The Catholic University of Korea, 222 Banpo-daero, Seocho-gu, Seoul, 06591 Korea; 3 Department of Ophthalmology, Seoul St. Mary’s Hospital, Seoul, Korea

**Keywords:** Cornea, Conjunctiva, Ocular surface, Povidone-iodine, Dry eye, Toxicity

## Abstract

**Background:**

We evaluated the toxicity of 5% (w/v) povidone-iodine (PI) applied to the ocular surface of rabbits.

**Methods:**

Twenty-three white rabbits were divided into four groups; these were a control group and three study groups in which the ocular surface was exposed to PI for different times. In control group, one drop of phosphate-buffered saline (PBS) was applied once for 10 min. In study groups, one drop of 5% (w/v) PI was topically applied once for 1 min, 3 min, and 10 min, and then the animals were observed for 7 days. The Schirmer test, Rose Bengal staining, corneal fluorescein staining and conjunctival impression cytology were performed on day 0, 3, and 7. After 7 days, the rabbits were sacrificed and conjunctiva and cornea were collected and evaluated by light and electron microscope. Immunofluorescence staining was also performed to detect mucin 5 subtype AC (MUC5AC).

**Results:**

The decrease in goblet cell density, reductions in MUC5AC level and histopathological and ultrastructural changes of conjunctiva and cornea were more prominent in the 5% (w/v) PI groups than the control group (*p* < 0.05). Moreover, these changes were more prominent when PI was applied for 3 and 10 min rather than 1 min (both *p* values < 0.05).

**Conclusions:**

5% (w/v) povidone-iodine caused damages to the ocular surface in a time-dependent manner. Therefore, we should be aware of that excessive PI exposure during ophthalmic procedures could be a pathogenic factor of dry eye syndrome after surgery.

## Background

Many factors may affect the ocular surface environment after cataract surgery and many patients have complained of dry eye and symptoms of irritations postoperatively. Khanal et al. reported that corneal sensitivity and tear physiology were significantly deteriorated immediately after phacoemulsification [1]. Li et al. suggested that misuse of eye drops is a pathogenic factor that causes dry eye after cataract surgery [2]. We showed that preservatives in eye drops such as benzalkonium chloride can damage the ocular surface in a dose-dependent manner [3]. And Ram et al. proposed that corneal denervation caused by surgical incision may also be a risk factor of dry eye after cataract surgery [4]. In our previous study, the duration of cataract surgical time was highly correlated with development of ocular surface damage such as goblet cell loss and tear film instability [5]. Also, light from the operating microscope was shown to exert phototoxic effects on the ocular surface and the tear film in vivo. Therefore, excessive light exposure during ophthalmic procedures may be one pathogenic factor causing dry eye syndrome after cataract surgery [6]. Of the many factors causing ocular surface damage during such surgery, we proposed that soaking in 5% (w/v) povidone-iodine (PI) may be one of the major causes a major cause of such damage. Recently, topical polyvinylpyrrolidone-iodine (PI) or chlorhexidine has become widely used as a disinfectant in most fields of ophthalmic surgery, for preoperative prevention of endophthalmitis [7, 8]. Previous studies have shown that application of 5% (w/v) PI to the cornea and conjunctiva effectively decreased the bacterial load of the ocular surface and the adnexae, and thus theoretically reduced the risk of postoperative endophthalmitis [9–12]. However, the product has an acidic pH (approximately 3.5), and preoperative PI has been shown to exert cytotoxic effects on the ocular surface epithelial cell [13, 14]. Thus, in the present study, we evaluated the effects of 5% (w/v) PI on the corneal and conjunctival surfaces of rabbit eyes, and ocular surface changes were compared based on PI exposure time. Although 5% (w/v) PI has been known to be toxic to the cornea, it has been proven effective to reduce bacteria on the ocular surface and widely used for preoperative preparation. So we decided to do this experiment with 5% (w/v) PI.

## Methods

All experimental procedures were performed according to the guidelines of the Association for Research in Vision and Ophthalmology (ARVO) as set out in the “Statement for the Use of Animals in Ophthalmic and Visual Research” and the ARRIVE Guidelines for reporting animal research, and approved by the institutional animal care and use committee of Catholic university of Korea (Yeouido-2012-17-01FC). Twenty-three New Zealand white male rabbits (DooYeol Biotech, Seoul, Korea) weighing between 2 and 2.5 kg were used in the study. The rabbits were housed in the individual mesh cages and given free access to standard laboratory food and water. They were housed in an air-conditioned room under a 12 h-light/12 h-dark cycle. No rabbit had either a corneal or conjunctival disorder. A commercially available 10% (w/v) PI solution was diluted with distilled water to 5% (w/v). Rabbits were randomly divided into four groups, with topical administration of phosphate-buffered saline (PBS) for 10 min in the control group (ten eyes), and topical administration of 5% (w/v) PI for 1 min, 3 min, and 10 min in groups 1, 2, and 3, respectively (12 eyes each) (Table 1). The PBS and 5% PI were instilled only single drop. A mixture of tiletamine and zolazepam (Zoletil®, Virbac, Carros, France) at 0.2 mg/kg was injected intramuscularly to immobilize the rabbits prior to performing the examinations, and proxymetacaine hydrochloride 0.5% (w/v) eye drops (Alcaine®, Alcon Laboratories Inc., Fort Worth, TX, USA) were applied topically. And before the collection of cornea and conjunctival tissues, all animals were killed by intraperitoneal injection of a lethal overdose of pentobarbital (100 mg/kg body weight). We conducted the experiments in the same way that we had previously conducted [3, 6]. And all experiments were conducted by the same standard techniques and under the same environmental conditions. The analysis and scoring of the specimens was done by a single masked investigator who did not know the information about each group. Although the number of samples was not large, the experiment was conducted with the smallest sample available for statistical analysis.

**Table 1:** Summary of control and study group

	Control group	Group 1	Group 2	Group 3
Numbers of eyes	10	12	12	12
Instilled solution and duration	PBS for 10 min	5% PI for 1 min	5% PI for 3 min	5% PI for 10 min

### The Schirmer test and rose Bengal staining

The Schirmer test was performed at baseline (day 0) and after PI instillation on day 3 and 7. The bent end of a standard Schirmer test strip (the Color Bar™; Eagle Vision, Memphis, TN) was placed in the lateral one-third of the inferior fornix. After 5 min, we measured the length of the moistened area of the strip.

Rose Bengal staining was performed on day 0 (immediately after PI treatment), 3, and 7. Two μL of 1% (w/v) Rose Bengal (Sigma, St. Louis, MO) were instilled into the conjunctival sac, and the extent of staining of the ocular surface was graded microscopically (Opmi6-CFC; Carl Zeiss AG, Jena, Germany) using the Van Bijsterveld grading system, which sums the staining grades of the nasal conjunctiva, cornea, and temporal conjunctiva; the maximum score is 3 per region and the maximum total score is thus 9 [15]. Although Rose Bengal stain pre-PI treatment data are required for an accurate comparison, it was not applied because all cornea and conjunctiva were considered healthy by slit lamp microscopy before instillation of PI, and Rose Bengal would have caused ocular surface damage. Thus, the analysis was made by comparison with the control group (Table 2).

**Table 2:** Summary of conducted examinations and number of eyes

	Examinations	Number of eyes
Day 0, 3, 7	Schirmer test Rose Bengal staining Fluorescein staining Conjunctival impression cytology (CIC)	6 eyes from control group and 8 eyes each from three PI groups
After day 7	Histological analysis Immunofluorescence staining	4 eyes from control group and 4 eyes each from three PI groups

### Corneal fluorescein staining

Fluorescein staining was performed on day 0 (immediately after PI treatment), 3, and 7. Two microliters of 1% sodium fluorescein were instilled into the lower fornix of the conjunctiva. The rabbit was allowed to blink several times to distribute the fluorescein evenly on the cornea. Two minutes later, corneal fluorescein staining intensity was also examined and graded under a portable slit-lamp microscope (Kowa SL-15; Kowa, Tokyo, Japan). We used the National Eye Institute/Industry grading scale (NEI) for corneal staining. There are five zones on the cornea (central, superior quadrant, inferior quadrant, nasal quadrant and temporal quadrant), and each zone is scored from 0 to 3(Grade 0: normal; Grade 1: mild; superficial stippling; Grade 2: moderate; punctuate staining including superficial abrasion of the cornea; Grade 3: severe; abrasion or corneal erosion, deep corneal abrasion or recurrent erosion). The maximum possible score was 15 [16].

### Conjunctival impression cytology

Conjunctival impression cytology (CIC) was performed on the upper bulbar conjunctiva after PI treatment on day 0, 3, and 7. CIC was performed only in the upper bulbar conjunctiva, as it is the most convenient site to reveal rabbit conjunctiva and collect a sufficient number of cells. A 4 × 3-mm mixed cellulose ester membrane (ADVANTEC®; Toyo Roshi Kaisha Ltd., Tokyo, Japan) was placed on the upper bulbar conjunctiva and constant pressure was applied for 5 s. Then it was lifted off, fixed in 10% (v/v) formaldehyde, and stained with hematoxylin and the periodic acid–Schiff (PAS) reagents. We counted the goblet cell density (GCD) and evaluated the morphology of the conjunctival epithelium using the Nelson system under a microscope (Axioskop 40; Carl Zeiss, Oberkochen, Germany) with a × 40 objective [17]. For goblet cell counting, we selected three different sections of each specimen randomly, and calculated the averages [cells/high power visual field (× 400)].

### Light microscopy of conjunctival tissue and Electron microscopy of corneal and conjunctival tissue

For light microscopic examination, the bulbar conjunctival tissues were collected on day 7 and fixed in 10% (v/v) formaldehyde. The dehydrated specimens were embedded in paraffin, cross-sectioned, and stained with hematoxylin and the PAS reagents. Then we examined the specimens under a microscope (Axioskop 40; Carl Zeiss, Oberkochen, Germany) with a × 40 objective. For transmission electron microscopic examination, the conjunctival tissues were fixed in 2% (v/v) paraformaldehyde/2.5% (v/v) glutaraldehyde, washed in 0.1 M phosphate buffer (pH 7.4), post-fixed in osmium tetroxide in phosphate buffer, and embedded in epoxy resin. Then they were cut into ultrathin sections, stained with uranyl acetate and lead citrate, and examined and photographed with a transmission electron microscope (TEM 1010; JEOL Ltd., Tokyo, Japan). For scanning electron microscopy (SEM), fixed corneal tissues were dehydrated in acetone, critical-point dried, mounted on metal stubs, and then sputtered with a 10 nm thick layer of gold in an ion sputter (JFC-1100; JEOL). Then we examined the specimens under a scanning electron microscope (Hitachi S-4700; Hitachi High-Technologies, Tokyo, Japan) at an acceleration voltage of 10 kV.

### Immunofluorescence staining for MUC5AC

MUC5AC has been identified as a major secretory mucin of conjunctival goblet cells and precorneal tear film. And many researchers have reported decreases in the ocular surface mucin expression including MUC5AC secreted by goblet cells in dry eye. We performed the immunofluorescence staining in the slides of the conjunctiva to detect the MUC5AC. The corneal specimens were served as one set of negative controls. The slides were deparaffinized and rehydrated in xylene (first) and ethanol (next), heated in boiling citrate buffer [10 mM citric acid, 0.05% (v/v) Tween-20 (pH 6.0)]; and treated (first) with 3% (v/v) H_2_O_2_, and (second) with peroxidase blocking solution [10% (v/v) FBS in PBS with 20 mM HEPES (4-(2-hydroxyethyl)-1-piperazineethanesulfonic acid)]. They were washed in PBS three times, and blocked with 1% (v/v) fetal bovine serum for 30 min at room temperature, followed by incubation for 12 h at 4 °C with a 1:100 dilution of mouse anti-human MUC5AC antibody (45 M1, catalog no. ab11335; Abcam, Cambridge, UK). After three times further washes in PBS, the slides were incubated with fluorescein isothiocyanate (FITC)-conjugated secondary goat anti-mouse IgG (Abcam) for 45 min at room temperature. Then we performed nuclear counterstaining with 0.5 μg/mL solution of Hoechst 33342 dye (Invitrogen, Carlsbad, CA), and examined the specimens under a fluorescence microscope.

### Statistical analysis

Statistical analysis was performed using SPSS version 18.0 (SPSS Inc., Chicago, IL). The statistical analysis on differences between individual groups based on time elapsed was conducted with repeated-measures analysis of variance (ANOVA) and Friedman’s test. The Rose Bengal test, corneal fluorescein staining score and Nelson scores of the various groups were compared using the Steel–Dwass test. The Tukey–Kramer test was used to compare goblet cell counts among the groups. A *p*-value less than 0.05 was considered to indicate statistical significance.

## Results

### Schirmer test values

There were no significant differences among the four groups at baseline, on day 3 and 7 (Table 3).

**Table 3:** The Schirmer test values of control and study groups

	Control group	Group 1	Group 2	Group 3	*p*-value
Baseline	16.17 ± 4.00	15.50 ± 3.90	17.67 ± 6.80	19.33 ± 4.60	0.882
Day 3	17.50 ± 3.40	15.72 ± 5.40	16.06 ± 4.60	16.00 ± 3.00	0.652
Day 7	19.50 ± 4.30	16.72 ± 3.70	15.50 ± 2.40	13.00 ± 5.40	0.772

### Rose Bengal staining

**Fig. 1:**
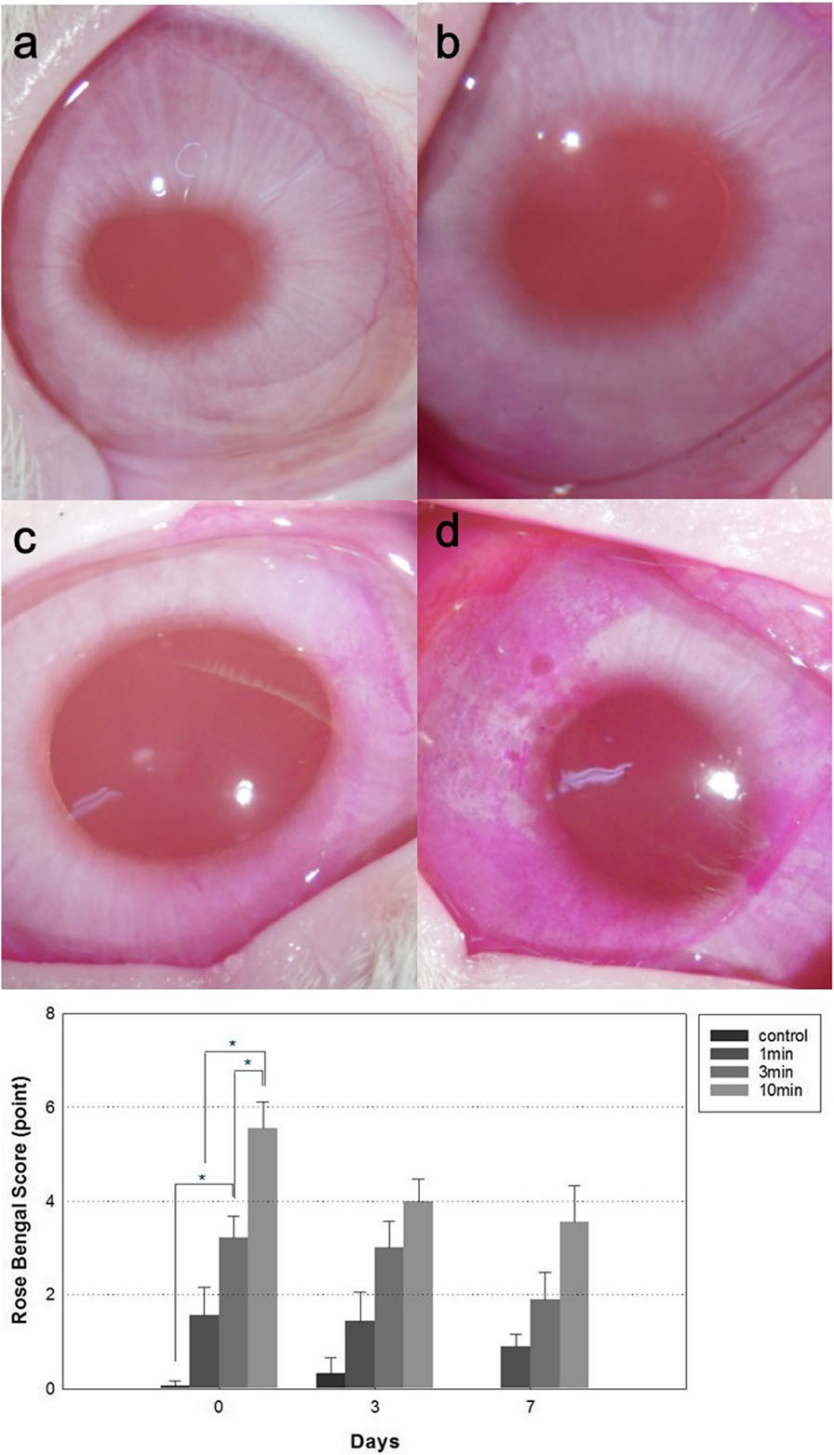
(above) Representative image samples stained with Rose Bengal in each group performed on day 0 (immediately after povidone-iodine (PI) treatment) (**a**) control group (**b**) 1-min PI group (**c**) 3-min PI group (**d**) 10-min PI group. (below) Comparison of Rose Bengal staining scores among the four groups. The Rose Bengal staining scores differed significantly between the control and 3-min PI group, the 3-min and 10-min PI groups, and the 1-min and 10-min PI groups on day 0. Data show means ± standard deviations (error bars). Day 0 indicates the day immediately after PI instillation. * *p* < 0.05 (Steel–Dwass test for the differences among the four groups)

The Rose Bengal staining scores differed among the four groups on day 0, 3, and 7 (on day 0; 0.08 ± 0.12 in the control group, 1.56 ± 0.60 in the 1-min PI group, 3.22 ± 0.46 in the 3-min PI group, and 5.56 ± 0.56 in the 10-min PI group; on day 3; 0.33 ± 0.33 in the control group, 1.44 ± 0.60 in the 1-min PI group, 3.00 ± 0.58 in the 3-min PI group, and 4.00 ± 0.47 in the 10-min PI group; on day 7; 0.00 ± 0.00 in the control group, 0.89 ± 0.26 in the 1-min PI group, 1.89 ± 0.59 in the 3-min PI group, and 3.56 ± 0.78 in the 10-min PI group, Fig. 1). However, significant differences were observed between the control and 3-min PI group, between the 3-min and 10-min PI groups, and between the 1-min and 10-min PI groups only immediately after instillation (*p* = 0.042, *p* = 0.040, *p* = 0.016, respectively, according to the Steel–Dwass test), and no significant differences were observed between the control and the 1-min PI group, and between the 1-min and the 3-min PI group (*p* = 0.155, *p* = 0.582, respectively, according to the Steel–Dwass test). There were no significant differences between the four groups on day 3 and 7 (*p* > 0.05 in the Steel–Dwass test). Overall, the area stained with Rose Bengal tended to increase as PI exposure time increased. And the Rose Bengal staining showed a tendency to improve with passage of time in all PI groups. However, only the 10-min PI group was statistically significant (*p* = 0.607 in control group, *p* = 0.905 in 1-min PI group, *p* = 0.513 in 3-min PI group, and *p* = 0.04 in 10-min PI group by Friedman’s test).

### Corneal fluorescein staining

**Fig. 2:**
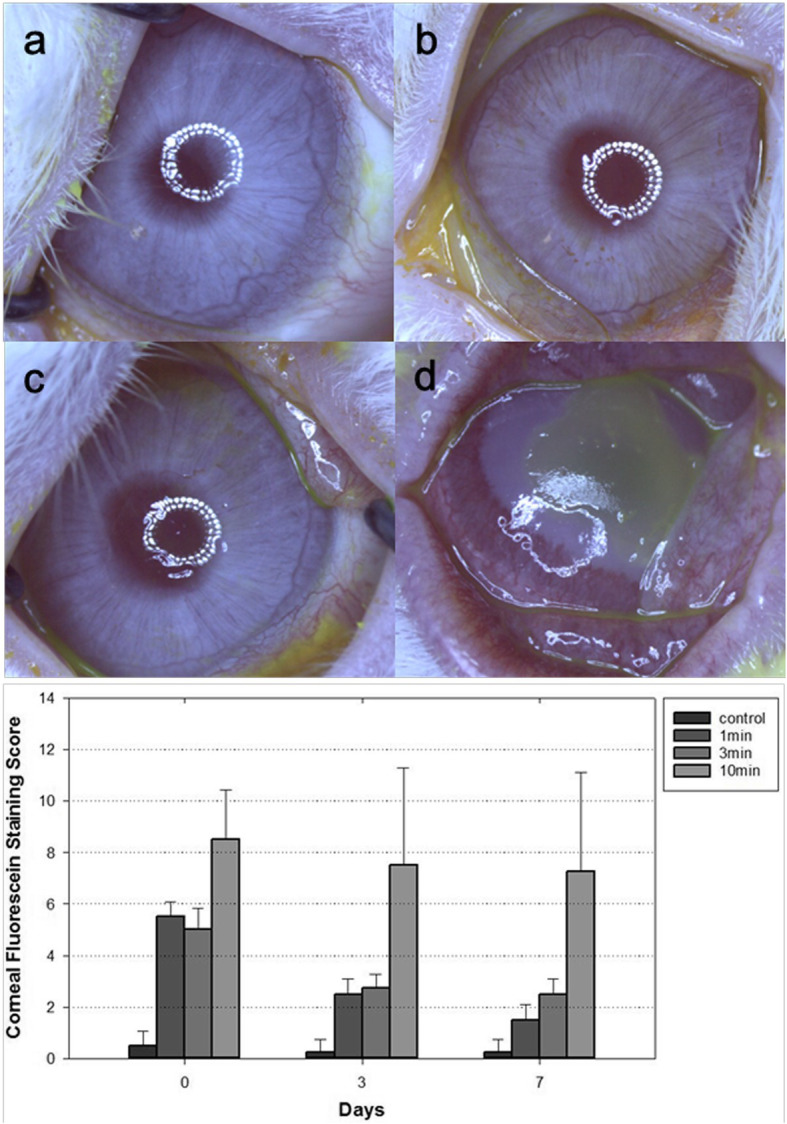
(above) Representative image samples stained with fluorescein in each group performed on day 7 (**a**) control group (**b**) 1-min PI group (**c**) 3-min PI group (**d**) 10-min PI group. (below) Comparison of corneal fluorescein staining scores among the four groups. 2b and 2c shows micropunctate or macropunctate staining of cornea in the 1-min and the 3-min PI groups. The 10-min PI group shows large epithelial defect on superficial cornea (**d**). No significant differences in the corneal fluorescein staining score at each time point (Steel–Dwass test for the differences among the four groups). Data show means ± standard deviations (error bars). Day 0 indicates the day immediately after PI instillation

The corneal fluorescein staining scores were counted of 0.5, 5.5, 5.0 and 8.5 each in the control group, 1-min, 3-min and 10-min PI group, respectively on day 0, 0.25, 2.5, 2.75 and 7.5 respectively on day 3, and 0.25, 1.5, 2.5 and 7.25 respectively on day 7 (Fig. 2). On day 3, some eyes of the 10-min PI group showed large epithelial defect on superficial cornea and no improvement until day 7 (Fig. 2d). The corneal fluorescein staining score showed significant improve with passage of time only in the 1-min and 3-min PI groups (*p* = 0.607 in control group, *p* = 0.038 in 1-min PI group, *p* = 0.022 in 3-min PI group, and *p* = 0.584 in 10-min PI group by Friedman’s test). The control and three PI groups showed no significant differences in the corneal fluorescein staining score at each time point (*p* > 0.05 by the Steel Dwass test). However, there were obvious corneal epithelial damages on some eyes of the 10-min PI group compared with the other groups.

### Conjunctival impression cytology

#### Goblet cell density (GCD)

The baseline GCD in the conjunctiva, before any instillation, was 92.3 ± 15 in the control group, 88.6 ± 11.2 in the 1-min PI group, 92.4 ± 13.9 in the 3-min PI group, and 93.7 ± 8.3 in the 10-min PI group. And when the GCD of the four groups were compared immediately after the PI instillation (on day 0), there were no significant differences between the four groups (*p* values of Tukey-Kramer test were 0.971, 0.996, 0.998, 0.976, 0.931 and 0.663 in the control vs 1-min PI group, control vs 3-min PI group, control vs 10-min PI group, 1-min vs 3-min PI group, 1-min vs 10-min PI group, 3-min vs 10-min PI group, respectively).

**Fig. 3:**
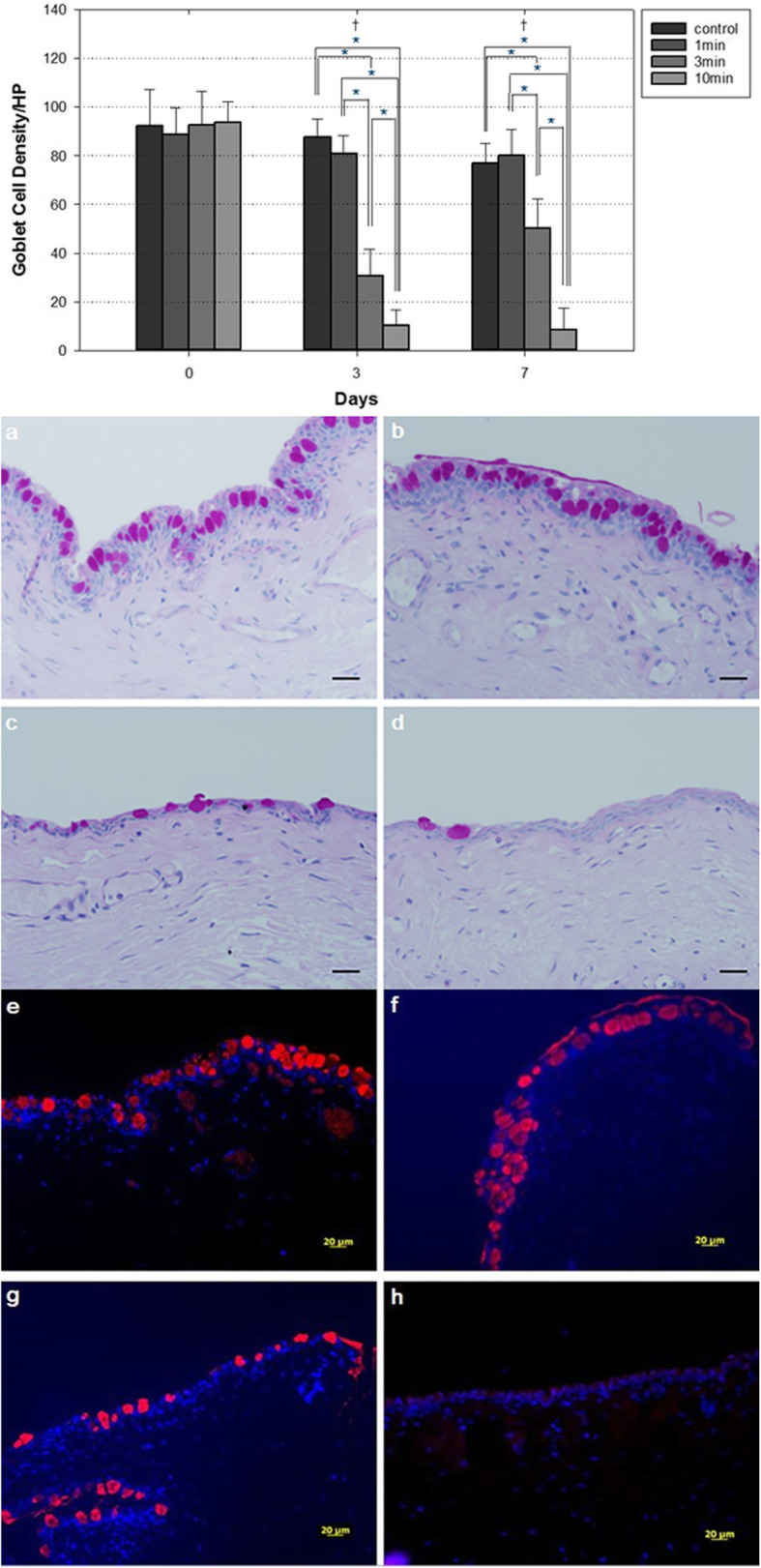
(top) Comparison of goblet cell density (GCD) among the four groups. Significant differences were noted between the control and 3-min PI group, the control and 10-min PI group, the 1-min and 3-min PI group, the 1-min and 10-min PI group, and the 3-min and 10-min PI group on day 3 and 7. Data are the means ± SDs (error bars). Day 0 indicates the day immediately after PI instillation. * *p* < 0.05 (Tukey–Kramer test for the differences among the four groups). (middle) Representative images obtained on histological examination of conjunctiva on day 7. **a** In the control group, the conjunctiva is composed of a single cuboidal basal cell layer and three-to-four layers of epithelial cells, with numerous interspersed goblet cells. **b** In the 1-min PI group, the conjunctival epithelium and goblet cell density are almost normal. **c** In the 3-min PI group, the conjunctival epithelium is thinner and the cuboidal basal cell layer is lost. The extent of the area stained by periodic acid-Schiff is reduced. **d** In the 10-min PI group, the conjunctival epithelium is even thinner and goblet cell numbers are decreased markedly. (Bar: 20 μm). (bottom) Representative images obtained on immunofluorescence staining of the conjunctiva on day 7. MUC5AC staining (red) was performed, and Hoechst 33342 was used for nuclear counterstaining (blue). **e** Many control group cells were detected in the anti-MUC5AC antibody conjunctiva stain. **f** Many cells also stained in the 1-min PI group. **g** The number of goblet cells stained by the anti-MUC5AC antibody decreased in the 3-min PI group. **f** No MUC5AC-positive cells are observed in the 10-min PI group. (Bar: 20 μm)

However, there were apparent significant differences on day 3 and 7 after PI instillation between the control and 3-min PI group, the control and 10-min PI group, the 1-min and 3-min PI group, the 1-min and 10-min PI group, and the 3-min and 10-min PI group (all *p* < 0.001 in the Tukey–Kramer test), whereas the control and 1-min PI group were not different significantly (*p* = 0.560 on day 3, *p* = 0.868 on day 7 in the Tukey–Kramer test). On day 3, the GCD was 87.7 ± 7.4 in the control group, 80.9 ± 7.4 in the 1-min PI group, 30.5 ± 11.2 in the 3-min PI group, and 10.3 ± 6.2 in the 10-min PI group. On day 7, the GCD was 76.9 ± 8.2 in the control group, 80.1 ± 10.8 in the 1-min PI group, 50.2 ± 12.1 in the 3-min PI group, and 8.6 ± 8.8 in the 10-min PI group (Fig. 3).

#### Severity of squamous metaplasia noted by conjunctival impression cytology

**Fig. 4:**
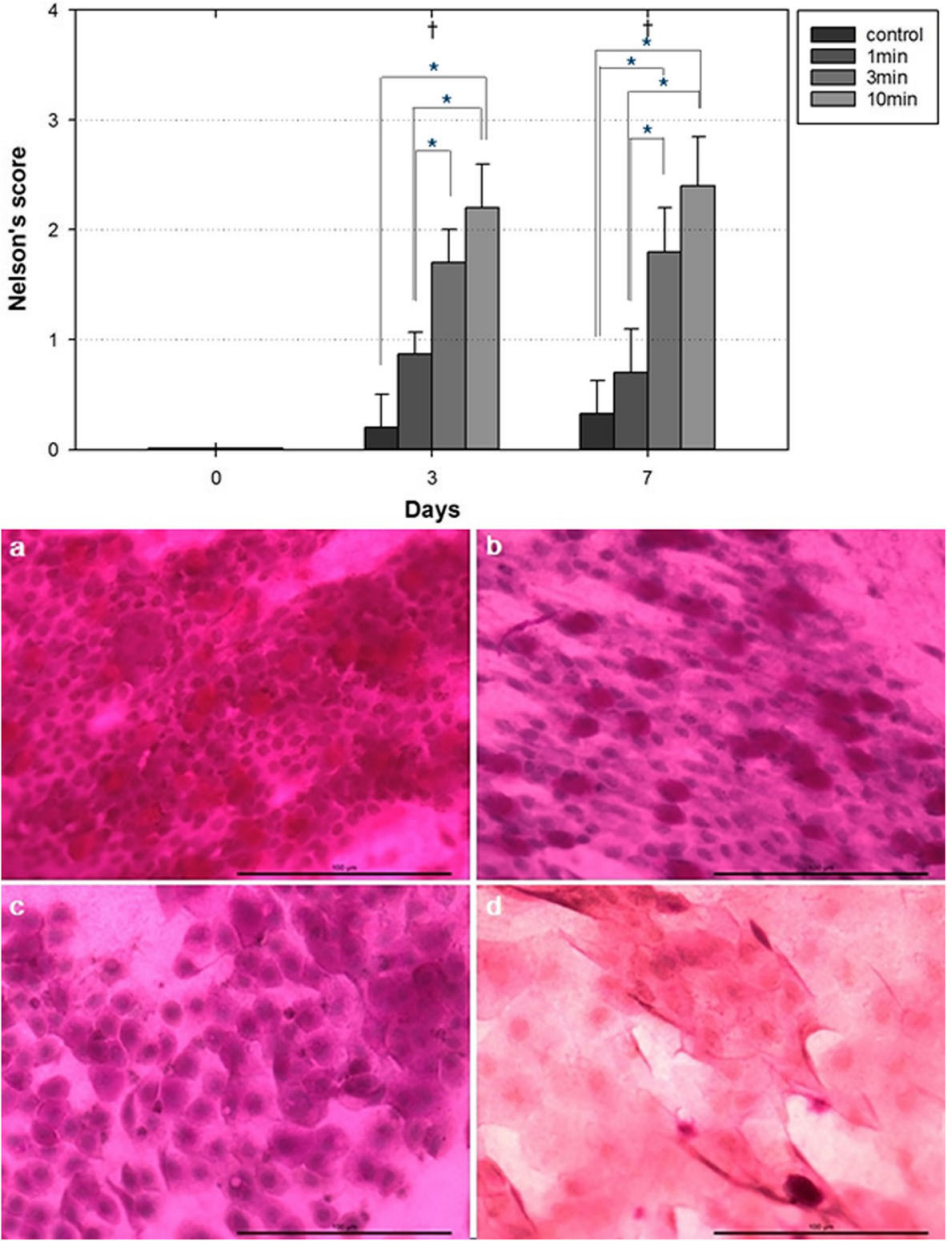
(above) Comparison of the severity of squamous metaplasia according to Nelson grading system among the four groups. Significant differences were noted between the control and 10-min PI group, the 1-min and 3-min PI group and the 1-min and 10-min PI group on day 3, and between the control and 3-min PI group, the control and 10-min PI group, the 1-min and 3-min PI group and the 1-min and 10-min PI group on day 7. Data show means ± SDs (the error bars). Day 0 indicates the day immediately after povidone-iodine instillation. * *p* < 0.05 (Steel–Dwass test for the differences among the three PI groups). (below) Representative images from conjunctival impression cytology of each group performed on day 7. **a** In the control group, the conjunctival impression cytological grade is 0. The epithelial cells are small and rounded. The nuclei are large, and stained with hematoxylin, at a nucleocytoplasmic ratio of 1:2. The abundant goblet cells are plump and oval with an intensely PAS-positive cytoplasm (400X). **b** In the 1-min PI group, the cytology grade is 0–1. The epithelial cells are slightly larger than control cells and more polygonal in shape. Goblet cells are abundant. **c** In the 3-min PI groups, the cytology grade is 2–3. The epithelial cells are slightly larger and more polygonal in shape. The nuclei are smaller, and have a nucleocytoplasmic ratio of between 1:2 and 1:3. Goblet cells numbers are markedly decreased. **d** In the 10-min PI group, the epithelial cells are larger and more polygonal. The nuclei are smaller and have a nucleocytoplasmic ratio of between 1:4 and 1:5. Goblet cells are scanty. Bar: 100 μm

When the severity of squamous metaplasia was compared using the Nelson grading system, the Nelson score tended to increase with PI exposure time on day 3 and 7. The Nelson score of the conjunctiva was 0.00 ± 0.00 in all group on day 0. On day 3, the Nelson score of the conjunctiva was 0.20 ± 0.30 in the control group, 0.87 ± 0.20 in the 1-min PI group, 1.70 ± 0.30 in the 3-min PI group, and 2.20 ± 0.40 in the 10-min PI group. On day 7, the Nelson score of the conjunctiva was 0.33 ± 0.30 in the control group, 0.70 ± 0.40 in the 1-min PI group, 1.80 ± 0.40 in the 3-min PI group, and 2.40 ± 0.45 in the 10-min PI group. The Nelson score was significantly different between the 1-min and 3-min PI group and between the 1-min and 10-min PI groups on day 7 (*p* = 0.013, *p* = 0.0031 by Steel–Dwass test), whereas no significant difference was observed between the control and 1-min PI group (*p* = 0.766). And this score showed a tendency to improve with passage of time in the 1-min, 3-min, and 10-min PI groups (*p* = 0.012, *p* = 0.001, *p* < 0.001 by Friedman’s test) (Fig. 4).

### Light microscopic examination of conjunctival tissue

Conjunctival cells prepared on day 7 were observed by light microscopy. Abundant goblet cells and multiple layers of epithelial cells were apparent in the control and 1-min PI group. However, the goblet cell counts decreased, and single layers of epithelial cells became more prevalent as the PI exposure time increased to more than 3 min (Fig. 3). These findings were similar to those obtained upon conjunctival impression cytology test.

### Immunofluorescence staining for MUC5AC of conjunctival tissue

Goblet cells were observed by immunofluorescence staining using an anti-MUC5AC antibody. Abundant MUC5AC was present in the conjunctival epithelia of the control and 1-min group. However, the extent of MUC5AC staining was reduced markedly in the 3-min and 10-min PI groups compared to the other groups (Fig. 3).

### Transmission electron microscopy (TEM) of conjunctival tissue

**Fig. 5:**
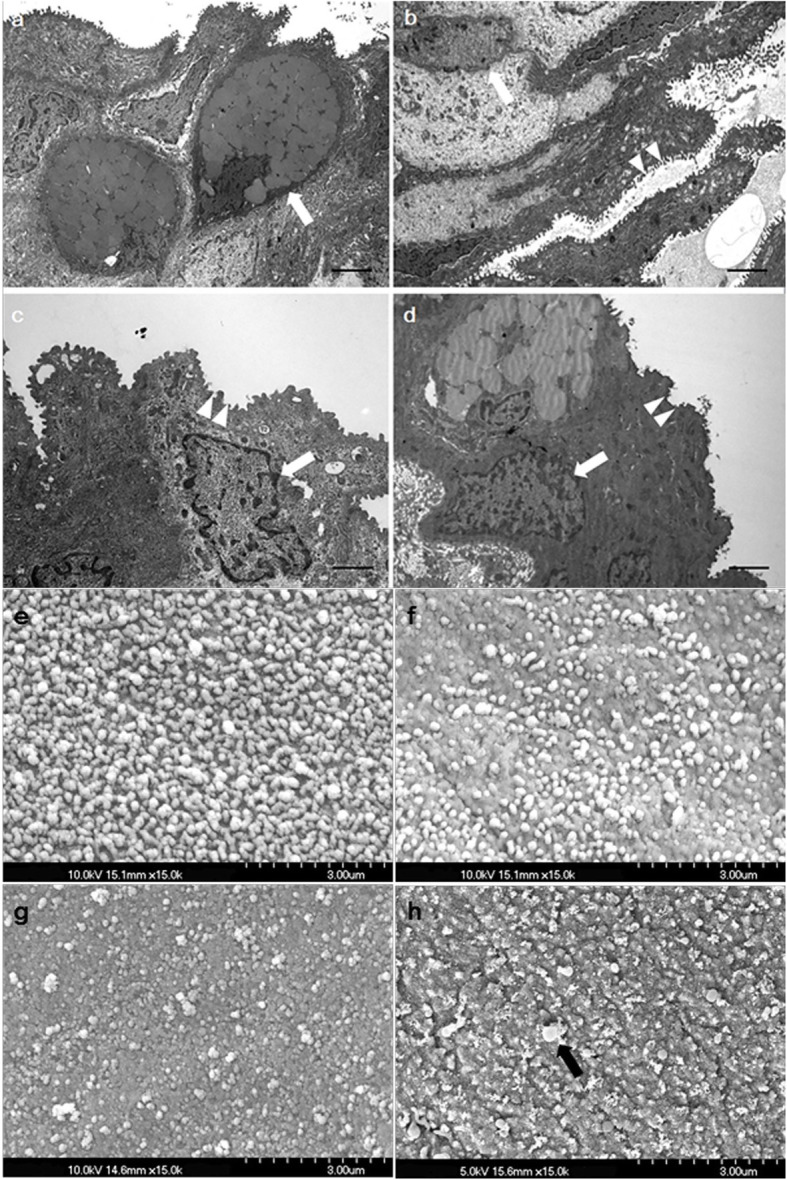
**a**-**d** Representative transmission electron microscopy (TEM) images of the conjunctiva and **e**-**h** scanning electron microscopy (SEM) images of the cornea on day 7. a,e: control group, b,f: 1-min PI group, c,g: 3-min PI group, d,h: 10-min PI group. TEM images revealing the conjunctival epithelial ultrastructure (**a**-**d**). Abundant microvillar structures on the surfaces of conjunctival epithelial cells (arrowhead) and goblet cells with many secretory granules (arrow) are observed in the control and 1-min PI group (**a**, **b**), whereas the extent of microvillar structure decreased, and apoptotic morphological changes (including nuclear fragmentation, condensation, and peripheral migration of chromatin) were shown in the other groups (**c**, **d**). (Bar: 2 μm). SEM images revealing the corneal epithelial structure (**e-h**). The superficial cells were intact with normal microvilli in the control group (**e**), whereas the flat surfaces with destroyed microvilli were shown in the all three PI groups. The longer the exposure time, the greater was the reduction in the number of microvilli (black arrow) (**f**-**h**)

Under the transmission electron microscope (TEM), abundant microvillar structures were evident on the surfaces of conjunctival epithelial cells of the control and 1-min PI group. In addition, goblet cells with many secretory granules, which play an important role in synthesis of MUC5AC, were observed (Fig. 5a, b). However in both the 3-min and 10-min PI groups, the extent of microvillar structure decreased, and apoptotic morphological changes including nuclear fragmentation, condensation and peripheral migration of chromatin were evident in conjunctival epithelial cells (Fig. 5c, d). Such ultrastructural disturbances were more severe in the 10-min PI group (Fig. 5d).

### Scanning electron microscopy (SEM) of corneal tissues

Scanning electron microscopy (SEM) showed that the superficial cells were intact with normal microvilli in the control group (Fig. 5e). However, SEM images showed corneal epithelial toxicity that the microvilli seemed to be injured in the all three PI groups. The longer the exposure time, the greater was the reduction in the number of microvilli (Fig. 5f, g, h).

## Discussion

Many studies have explored ocular surface damage associated with cataract surgery [1, 2, 4–6, 18–22]. Some factors are well-known to be associated with postoperative dry eye. These include corneal damage caused by ultrasound used during cataract surgery, topical anesthesia, eye drops containing preservatives such as benzalkonium chloride, corneal desensitization caused by corneal incision, surgical trauma, inflammatory responses producing chemicals such as free oxygen radicals, and exposure to light from microscopes [1, 2, 4–6, 18–22]. In clinical practice, some ophthalmologists have detected corneal damage immediately after preoperative PI instillation. Thus, we hypothesized that PI instillation might be pathogenic factor of dry eye syndrome in terms of inducing ocular surface damage in patients undergoing ophthalmic surgery. The purpose of our study was to evaluate the effects of 5% (w/v) PI on the ocular surfaces and identify possible pathognomonic factor triggering ocular surface damage after cataract surgery.

We evaluated the effects of 5% (w/v) PI applied for various exposure times, after single instillation onto the ocular surface of normal rabbits, by performing the Schirmer test, Rose Bengal staining, corneal fluorescein staining, conjunctival impression cytology and biopsy. We compared the changes of ocular surface among control group and study groups exposed to 5% (w/v) PI for 1, 3, and 10 min. The Rose Bengal staining scores were significantly increased immediately after PI instillation as the PI exposure time increased although the Schirmer test results were not different among the four groups. And on conjunctival impression cytology, the GCD was decreased and the Nelson score was increased with increasing exposure time. The GCD and Nelson score were significantly different between the 1-min and 3-min PI group and the 1-min and 10-min PI group. Conjunctival histology performed on day 7 revealed similar results. The conjunctival epithelial region of MUC5AC staining was reduced markedly in the 3-min and 10-min PI groups compared to the other groups. And SEM-based histological analyses showed loss of microplicae of superficial cornea. This corneal epithelial cell damage is also the reason for the positive staining in the fluorescein. We noted that exposure time-dependent pathophysiological ocular surface changes were similar to those seen in the dry eye syndrome; these included the reduction of GCD, an increase of conjunctival epithelial cell size, squamous metaplasia of conjunctiva and damaged superficial layer of the cornea.

The mucin and the microplicae play a role in tear film adhesion and stabilization to the corneal surface. And the microplicae are also important for the attachment of mucin to the cornea. Decreased production of MUC5AC, as well as changes in membrane-associated mucins also leads to loss of microplicae [23–26]. The functions of mucous layer play a vital role in the stability of the tear film, converting the hydrophobic corneal epithelium to be hydrophilic and lubricating the ocular and palpebral surfaces. Thus, the damages of microplicae and decreased mucin production induced by PI interfere the formation of the innermost mucous layer of the tear film, and would lead to less retention of fluid even with functional lacrimal glands.

It is important to define the ocular surface toxicity caused by PI because of the recent spotlight cast on ocular surgery-associated dry eye syndrome. PI has been reported to be cytotoxic to the eye. Jiang et al. found that severe corneal epithelial damages were developed after PI instillation into the conjunctival sac, and significant corneal edema was observed after PI injection into the anterior chamber [3]. MacCrae et al. studied rabbit corneas after PI application, and noted moderate transient corneal edema at 5 min, which was resolved 3 h later [14]. Whitacre and Crockett assessed the toxicity of intravitreal PI by injecting 0.1 mL amounts of PI solutions at 0.05, 0.5, and 5% (all w/v) into the vitreous cavity [27]. One of 10 eyes injected with 0.05% PI had iritis, intraretinal hemorrhage and mild retinal necrosis, whereas all four eyes injected with 5% (w/v) PI had dense cataracts and full-thickness retinal necrosis. Apart from the direct toxicity of topical PI for the ocular surface, both contact dermatitis and keratoconjunctivitis sicca have been (rarely) reported [28, 29]. Therefore, intraocular contamination with PI is of concern. To the best of our knowledge, few studies have addressed the cytotoxicity of PI for the ocular surface, especially the effect of PI on conjunctival goblet cell function. And unfortunately, PI exposure times were not compared; all data were compared to only those of the control group. Although ocular toxicity of PI may increase as the exposure time becomes longer, few studies have sought to clearly define an appropriate exposure time.

It is clear that, in vivo, periocular preparation with PI alone (even for a long time) may not completely eradicate all types of microorganism that give rise to postoperative endophthalmitis. And as the results of present study show, the longer the PI exposure time, the more severe the ocular surface damages, so in order to minimize ocular surface toxicity, it would be better to use PI an optimum exposure time along with other prophylactic antibiotics or bactericidal agents that supplement the disinfective effect. In our present study, PI exposure for 1 min was not associated with any microscopically apparent damage to conjunctival epithelial cells, although corneal and conjunctival epithelial cells that were no longer adequately protected by tear films stained with Rose Bengal to a greater extent than control group. Upon conjunctival impression cytology, it was remarkable that no significant difference was apparent between the control and 1-min PI group. Thus, if the antimicrobial effect of 1 min of PI exposure is as effective as that afforded by 3 min of PI exposure, the optimum PI exposure time affording maximum eradication of microorganisms capable of causing endophthalmitis, but without causing ocular surface toxicity, should be 1 min rather than the 3 min of the previous guidelines.

The present study had several limitations. The follow-up period (7 days) was short, and the sample size was small, meaning that possible reversibility of ocular surface toxicity was not observed. The GCD of the 3-min group tended to recover on day 7. Our results indicate that long-term instillation of PI causes acute injury to the ocular surface and may cause chronic injury or induce dry eye. Nevertheless, as the 10-min group, unlike the 3-min group, showed no signs of recovery, further study is required to determine the possibility of chronic conjunctival injury following long-term PI instillation, to explore whether and when damaged ocular surfaces can recover. And in the present study, we applied just one drop of 5% PI for different exposure time. But nowadays, many authors recommend multiple applications of PI for a short exposure time. Therefore, we need more research to determine the effect of multiple short exposures versus one long exposure. Unfortunately, we could not check tear break up time for technical reasons and equipment-related problems. Although we could not demonstrate whether the decrease in GCD and MUC5AC directly caused dry eye, one study suggested a correlation between MUC5AC expression and dry eye clinical test results such as tear break up time [30]. Expression of conjunctival MUC5AC and the squamous metaplasia in CIC are closely correlated with tear break up time as a dry eye severity indicator [30, 31]. Therefore, based on these studies, we consider that the laboratory tests implemented in our study might supersede tear break up time. Use of PI may be a pathogenic factor causing postoperative dry eye resulting from the decrease in GCD and MUC5AC stain. However, we will measure tear break up time for confirmation in our next human clinical trial.

## Conclusions

In conclusion, we have shown that longer exposure times with 5% (w/v) PI increasingly damaged the cornea and conjunctiva of normal rabbits (as shown by Rose Bengal staining and fluorescein staining); decreased the GCD, and triggered development of squamous metaplasia, as revealed by impression cytology and corneal and conjunctival histology. We could not confirm whether PI toxicity triggers not only acute injury but also chronic injury. So, further clinical study is required to clarify the relationship between PI and ocular surface toxicity. And also, we evaluated that such an instillation of PI might be pathogenic in terms of inducing ocular surface damage in patients undergoing ophthalmic surgery. Therefore, the toxicities of PI to ocular surface could be a possible pathogenic factor triggering dry eye syndrome after ophthalmic surgery.

## Data Availability

All data are available from the corresponding author on reasonable request.

## Abbreviations

CIC: Conjunctival impression cytology
GCD: Goblet cell density
MUC5AC: Mucin 5 subtype AC
PAS: Periodic acid–Schiff
PBS: Phosphate-buffered saline
PI: Povidone-iodine
SEM: Scanning electron microscopy
TEM: Transmission electron microscope
